# Ticagrelor versus Clopidogrel in Patients with Severe Renal Insufficiency Undergoing PCI for Acute Coronary Syndrome

**DOI:** 10.1155/2022/6476777

**Published:** 2022-07-31

**Authors:** Yunxian Chen, Shaowen Tu, Zhixin Chen, Jue Xia, Baofeng Chen, Jinfeng Chen, Jiarong Liang, Xiangyang Liu, Liangqiu Tang

**Affiliations:** Department of Cardiology, Yue Bei People's Hospital, Shantou University Medical College, Shaoguan, China

## Abstract

**Background:**

Current guidelines recommend the use of potent antiplatelet agents in patients undergoing percutaneous coronary intervention (PCI) following an acute coronary syndrome (ACS). However, data about optimal platelet inhibition in severe renal insufficiency patients are scarce. The purpose of this study is to determine if ticagrelor is more effective than clopidogrel in patients with ACS and severe renal insufficiency treated with PCI.

**Methods:**

We retrospectively enrolled patients with ACS and severe renal insufficiency (eGFR ≤ 30 ml/min·1.73 m^2^ or dialysis) who underwent PCI at our hospital between January 2015 and March 2020. We used the adjusted Cox proportional hazards models to analyze the 1-year outcome endpoints, including the primary endpoint (the composite of cardiovascular death, recurrence of MI, or nonfatal ischemic stroke), death from any cause, and bleeding events (Bleeding Academic Research Consortium, BARC criteria).

**Results:**

A total of 276 patients with ACS and severe renal insufficiency who were treated with PCI with ticagrelor (*n* = 108) or clopidogrel (*n* = 168) were included in the study. After adjustment, there was no statistical difference in risk of the primary endpoint (HR, 0.78; 95% CI, 0.46–1.33; *P*=0.367) and death from any cause (HR, 0.86; 95% CI, 0.38–1.89; *P*=0.708) in the ticagrelor group against the clopidogrel group. However, the risk of total bleeding was significantly higher in the ticagrelor group (HR, 3.01; 95% CI, 1.81–5.62; *P*=0.01). Subgroup analysis according to the confounders did not identify any significant subgroup heterogeneity.

**Conclusion:**

Ticagrelor did not improve the major adverse cardiovascular events and all-cause mortality when compared to clopidogrel, but significantly increased the risk of bleeding in Chinese patients with ACS and severe renal insufficiency undergoing PCI.

## 1. Introduction

Chronic kidney disease (CKD) affects between 20% and 40% of patients hospitalized with the acute coronary syndrome (ACS), and these individuals have a greater death rate than those with normal renal function [1]. Studies have shown that percutaneous coronary intervention (PCI) improves the prognosis of patients with ACS and CKD, but patients with renal insufficiency have a higher risk of thrombosis and bleeding after the procedure [2]. Current guidelines recommend dual antiplatelet therapy (DAPT) with aspirin and P2Y12 receptor inhibitors for 12 months after PCI in patients with ACS [3].

Ticagrelor or prasugrel, both of which are potent oral P2Y12 receptor inhibitors, are recommended for the treatment of patients with ACS and CKD due to their rapid onset of action and high rate of platelet inhibition compared to clopidogrel [3–5]. Clopidogrel has a delayed onset of effect and wide interindividual variability in biological efficacy leading to a 40% rate of high on-treatment platelet reactivity (HTPR) [6, 7]. Importantly, CKD patients have a greater risk of HTPR than the overall population, which was linked to thrombotic events and death [8, 9]. Ticagrelor is more powerful and reproducible in its platelet inhibition than clopidogrel, even in CKD patients [10–12].

Numerous studies have demonstrated that ticagrelor improves clinical outcomes such as all-cause mortality and major cardiovascular adverse events in patients with ACS and CKD when compared to clopidogrel [13–17]. However, it has also been shown that ticagrelor is not superior to clopidogrel and may increase the risk of bleeding [17–19]. Since most of the previous studies were conducted in patients with mild to moderate renal insufficiency, the efficacy and safety of DAPT with potent P2Y12 inhibitors in patients with severe renal insufficiency is unclear [15, 20]. This study was designed to evaluate the 1-year clinical outcomes of ticagrelor vs. clopidogrel in patients with severe renal insufficiency (eGFR ≤ 30 ml/min·1.73 m^2^ or dialysis) undergoing PCI, including cardiovascular death, death from any cause, MI, stroke, and bleeding events.

## 2. Methods

### 2.1. Study Design and Patient Population

This was a single-center retrospective study. We consecutively selected patients with ACS and severe renal function (eGFR < 30 ml/min·1.73 m^2^), including patients on hemodialysis treatment who underwent successful PCI in our hospital between January 2015 and March 2020. We excluded all patients on anticoagulant therapy at discharge, patients with missing creatinine measurements, patients not receiving dual antiplatelet therapy at discharge, and patients lost to follow-up within 1 year. Patients were divided into the ticagrelor group or the clopidogrel group according to the dual antiplatelet regimen during hospitalization and after hospital discharge. All patients' baseline characteristics, past medical history, clinical test results, hospital medications, and angiographic data should be documented. The Ethics Committee of Yuebei People's Hospital approved the study protocol, and all study subjects provided informed consent (Registration Number: ChiCTR2100043135). All procedures were carried out in accordance with the applicable guidelines and regulations.

### 2.2. Definitions

ACS, including unstable angina, non-ST-segment elevation myocardial infarction, and ST-segment myocardial infarction, were defined according to the diagnostic criteria established by the European Society of Cardiology [5]. According to the Bleeding Academic Research Consortium (BARC) classification [21], we defined mild to moderate bleeding as BARC 1 or 2 and severe bleeding as BARC 3 or 5. The estimated glomerular filtration rate (eGFR) was calculated from creatinine measurements on admission and using the Chronic Kidney Disease Epidemiology Collaboration equation [22]. Cardiovascular death was defined as death because of AMI, heart failure, cardiogenic shock, ventricular arrhythmia, or cerebrovascular events.

### 2.3. Interventional Procedures and In-Hospital Medications

PCI procedures were performed by three experienced surgeons, and all patients were implanted with a Firebird2 coronary rapamycin-eluting cobalt-based alloy stent (MicroPort Scientific Corporation, China) [23]. All patients underwent revascularization of the culprit's vessel. The decision to perform complete revascularization depended on the site of the vascular lesion, its severity, the patient's general condition, and the surgeon's strategy. Because of the health insurance policy, there were only two new oral antiplatelet drugs at our hospital, clopidogrel and ticagrelor, and no prasugrel. Clinicians chose different antiplatelet agents for treatment based on guidelines, personal experience, and the patient's condition. Before the intervention, all patients received antiplatelet agents, including aspirin 300 mg loading dose (LD) and clopidogrel 300–600 mg LD or ticagrelor 180 mg LD. Following the intervention, the patients were given aspirin 100 mg once daily indefinitely, as well as clopidogrel 75 mg once daily or ticagrelor 90 mg twice daily for at least a year. Other drugs, such as glycoprotein IIb/IIIa inhibitors, antithrombotic drugs, ACEI/ARB, beta-blockers, and so on, were chosen based on the clinical situation of the patient.

### 2.4. Study Endpoint

During a 1-year follow-up period, adverse clinical outcomes were recorded. We used major adverse cardiovascular events (MACEs, the composite of cardiovascular death, recurrence of MI, or nonfatal ischemic stroke) as a primary endpoint. The secondary endpoint included the individual components of the primary outcome described separately as well as death from any cause and bleeding events (BARC classification).

### 2.5. Statistical Analysis

Continuous data were expressed as a median ± standard or an interquartile range (IQR), while categorical data were expressed as percentages. The variables were compared using the chi-square tests or Fisher's exact tests (categorical variables), the Student's *t* (continuous variables), and Kruskal–Wallis (skewed distribution) tests, respectively. Kaplan–Meier analysis was used to assess the event-free status of the patients using the log-rank test. Multivariable Cox regression analyses were adopted to assess the independent association between DAPT regimens and 1-year outcomes after adjusting for clinical characteristics. Subgroup analyses were stratified by some relevant effect covariates. All the analyses were performed with the statistical software packages *R* (https://www.R-project.org, The *R* Foundation) and Free Statistics software version 1.3. A two-tailed test was performed and p 0.05 was considered statistically significant.

## 3. Results

### 3.1. Population and Baseline Characteristics

**Figure 1:**
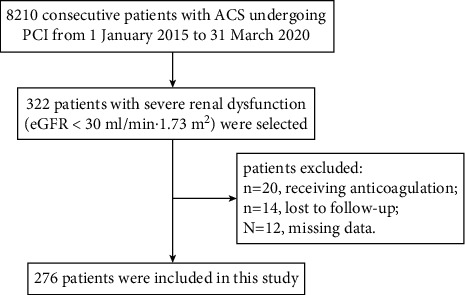
Flowchart of patient selection.

Between January 2015 and March 2020, our hospital treated 8210 consecutive patients with ACS who underwent PCI. In total, 322 of these patients had severe renal function (eGFR < 30 ml/min·1.73 m^2^). We excluded 20 patients who were on anticoagulant therapy at discharge, 12 patients with missing dates, and 14 patients lost to follow-up, resulting in a study population of 276 patients. The flow chart of the study patients' selection is shown in Figure 1. Enrolled patients were divided into 2 groups: ticagrelor group (*N* = 108) and clopidogrel group (*N* = 168). There was no statistical difference between the 2 groups on age, sex, diagnosis at discharge, traditional cardiovascular risk, previous medical history, dialysis, creatine clearance, treated vessel, or medications at admission. The baseline characteristics of all participants are given in Table 1.

### 3.2. Cardiovascular Outcomes

**Figure 2:**
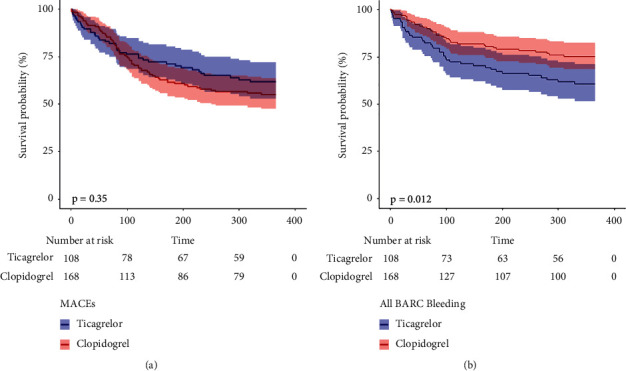
Kaplan–Meier curve for the 1-year primary endpoint (a) and total BARC bleeding (b) between clopidogrel and ticagrelor group.

**Figure 3:**
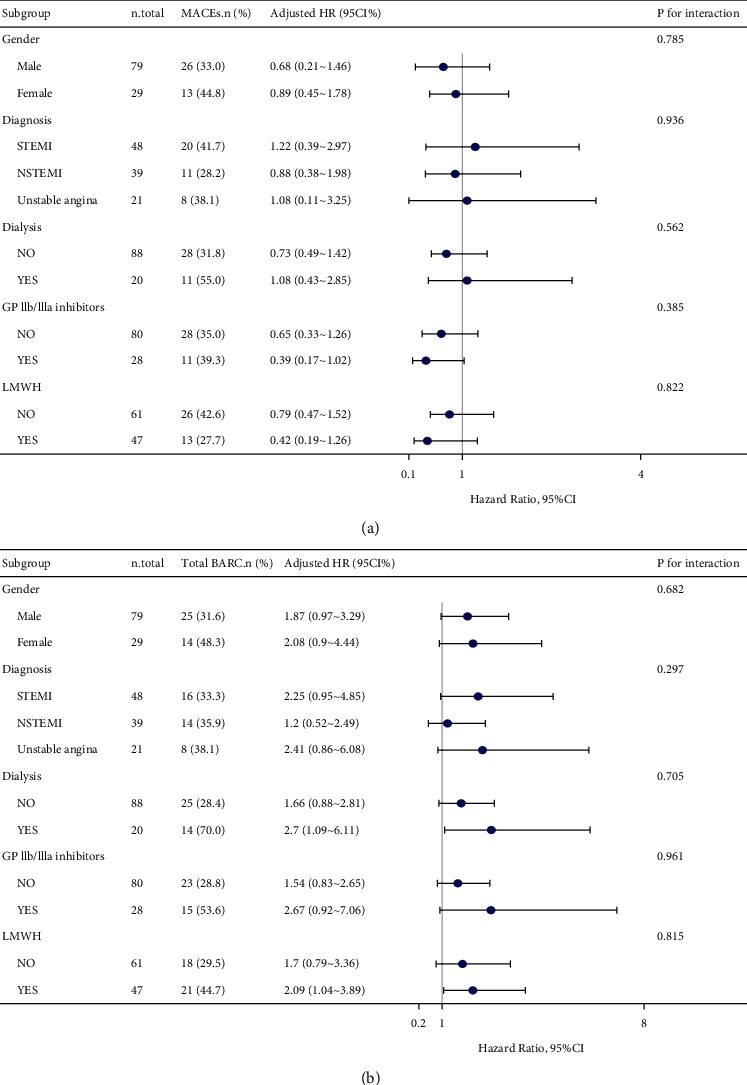
Forest plot illustrating the adjusted HR for primary outcomes (a) and total BARC bleeding (b) stratified on confounding factors (including gender, ACS staging, hemodialysis, use of glycoprotein IIb/IIIa inhibitors, and low-molecular heparin). STEMI, ST-elevation myocardial infarction; NSTEMI, non-ST-elevation myocardial infarction; LMWH, low-molecular-weight heparin.

During the 1-year observation period, 39 (36.1%) patients in the ticagrelor group and 69 (41.1%) patients in the clopidogrel group met the primary endpoint. After adjustment, there were no significant differences in the risk of the primary endpoint (HR, 0.78; 95% CI, 0.46–1.33; *P* = 0.367), death from any cause (HR, 0.86; 95% CI, 0.38–1.89; *P* = 0.708), cardiovascular death (HR, 0.80; 95% CI, 0.35–2.12; *P* = 0.521), recurrent MI (HR, 0.72; 95% CI, 0.31–2.36; *P* = 0.485), or stroke (HR, 0.46; 95% CI, 0.24–3.07; *P* = 0.172) between the clopidogrel and ticagrelor groups in any of the three extended multivariable Cox models (Table 2). Kaplan–Meier analysis showed no difference between the two groups in the 1-year primary endpoint (Log-rank test: *P* = 0.35, Figure 2(a)). Subgroup analyses were performed according to confounding factors including gender, ACS staging, hemodialysis, use of glycoprotein IIb/IIIa inhibitors, and low-molecular heparin, and we did not observe any significant interactions in the subgroups (*P* value for interaction >0.05 for all, Figure 3(a)).

### 3.3. Bleeding

During the 1-year observation period, 39 (36.1%) patients in the ticagrelor group and 37 (22.0%) patients in the clopidogrel group occurred any bleeding events. After adjusted, ticagrelor was associated with a significantly higher risk of total BARC bleedings (HR 3.01, 95% CI 1.81–5.62, *P*=0.010), BARC 1 or 2 bleedings (HR 3.14, 95% CI 1.52–5.76, *P*=0.018) and BARC 3 or 5 bleedings (HR 2.87, 95% CI 1.12–7.03, *P*=0.045) when compared to clopidogrel (Table 3). Kaplan–Meier curve showed a significantly higher risk of total BARC bleeding in the ticagrelor group than in the clopidogrel group (Log-rank test: *P*=0.012, Figure 2(b)). Subgroup analysis also did not identify any significant subgroup heterogeneity (*P* value for interaction >0.05 for all, Figure 3(b)).

## 4. Discussion

Patients with CKD have a higher incidence of ACS, as well as a higher risk of thrombosis and bleeding, with worse outcomes than the general population [24]. The clear recommendation regarding the selection of P2Y12 inhibitors in CKD patients including those with severe CKD (eGFR < 30 mL/min) is unavailable [5, 25]. We conducted this study to compare the 1-year clinical outcomes between the clopidogrel and the ticagrelor groups in patients with severe renal insufficiency undergoing PCI. The main findings of our study were that ticagrelor did not improve the primary endpoint (the composite of cardiovascular death, recurrence of MI, or nonfatal ischemic stroke) and all-cause mortality when compared to clopidogrel, but did increase bleeding events in these patients.

### 4.1. Effect of Ticagrelor and Clopidogrel on Cardiovascular Outcomes in ACS Patients with Severe Renal Insufficiency

Numerous studies, including the well-known PLATO randomized controlled clinical trial, have demonstrated that ticagrelor reduces the risk of vascular death, all-cause death, myocardial infarction, stent thrombosis, or stroke in patients with ACS receiving or not receiving PCI [13, 15, 26]. These findings are supported by some basic research. Patients with ACS and CKD have a high thrombotic risk related to 3 main factors: alteration of the coagulation cascade, endothelial injury, and platelet alteration [4]. Patients with CKD have greater ADP-induced platelet aggregation, and the degree of reduction in clopidogrel response increases with renal insufficiency [7]. From a biological point of view, ticagrelor has a more rapid onset of action and induces a more potent and reproducible platelet inhibition than clopidogrel [12, 27].

There may be several reasons why our findings differed from these studies. First, our study population is different from the current studies in that we enrolled patients with an estimated glomerular filtration rate of less than 30 ml/min·1.73 m^2^, whereas most other studies enrolled patients with a creatinine clearance of less than 60 ml/min [4, 16, 17]. Furthermore, in some other trials, the number of stage 4 CKD and end-stage kidney failure was limited. For example, in the PLATO trial, the proportion of CKD patients was 21.3%, whereas only 214 patients had a calculated clearance of less than 30 mL/min and dialysis was excluded [13]. While in PLATO more than one-third of patients were treated conservatively, all patients in our study were treated with PCI. Second, it is possible that Asians have a lower rate of thrombosis and fewer ischemic events than Western patients [28]. Several clinical studies from Asia have shown that ticagrelor is not superior to clopidogrel in patients with ACS with normal renal function and may increase the risk of bleeding [18, 19, 29, 30]. In a separate endpoint analysis, our data suggested that cardiovascular death (4% absolute risk difference) and myocardial infarction (4.4% absolute risk difference) were lower in the ticagrelor group than in clopidogrel, though this difference was not statistically significant. This could imply that ticagrelor may still reduce cardiovascular adverse events in ACS patients with severe renal insufficiency. However, we were unable to reach this conclusion at this time. This was because ticagrelor patients may be in the better physical condition and so had a better prognosis. More prospective randomized controlled trials are needed to validate this conclusion. In our study, the 1-year cardiovascular events rate was high for both ticagrelor and clopidogrel. In addition, patients suffering from severe renal insufficiency have a poor prognosis and are more likely to die from infection, heart failure, respiratory failure, or other complications [31]. This suggests that antiplatelet therapy may only be part of the treatment for ACS patients with severe renal insufficiency.

### 4.2. Effect of Ticagrelor and Clopidogrel on Bleeding Outcomes in ACS Patients with Severe Renal Insufficiency

Our study showed a high risk of bleeding in patients with severe renal insufficiency and that ticagrelor increased the risk of bleeding in patients compared to clopidogrel. This was consistent with the findings of other Asian studies. In Korean acute coronary syndrome patients intended to receive early invasive management, standard-dose ticagrelor as compared with clopidogrel was associated with a higher incidence of clinically significant bleeding [19]. Among real-world Chinese patients with ACS treated by PCI, ticagrelor only showed superior efficacy in patients with low bleeding risk but lost its advantage in patients with moderate-to-high bleeding potential [32]. A review found that ticagrelor and clopidogrel had comparable efficacies in East Asian patients with ACS. Ticagrelor therapy also displays some side effects including an increased risk of major bleeding [29]. It was crucial to note that our study found that ticagrelor increased the risk of mild to moderate hemorrhage more than severe bleeding.

Studies have shown that low body weight, anemia, and chronic kidney disease were risk factors for major bleeding after ticagrelor therapy [33, 34]. In the real world, patients with severe renal insufficiency often have chronic anemia, so these patients are at a much higher risk of bleeding [17]. It should be underlined that significant bleeding events have an impact on total patient mortality, and this is especially true for ACS patients undergoing PCI. Some studies in recent years have shown that early de-escalation, including de-escalation from a potent P2Y12 inhibitor to clopidogrel or low-dose ticagrelor, was an effective strategy for ACS treatment, resulting in fewer bleeding events without increasing ischemic events [35–37]. In ACS patients with severe renal insufficiency, early de-escalation of antiplatelet therapy may be considered as an alternate strategy.

### 4.3. Limitations

This study has several limitations. First, it was a retrospective study and some confounding bias was difficult to exclude. Second, all data were collected from a single medical center. Due to the small sample size, we did not perform statistical adjustments including propensity scores matching, which may lead to less representative results. Third, there was a possibility of selection bias in our study since clinical practitioners may choose a less potent antiplatelet drug for patients with bleeding tendencies. Lastly, we categorized patients based on their DAPT regimen throughout their hospitalization and did not take into account differences in DAPT regimen duration. There was no investigation into whether patients changed antiplatelet medicines following a cardiovascular or bleeding incident.

## 5. Conclusion

Our study shows that the use of ticagrelor did not improve the composite of cardiovascular death, recurrence of MI, or nonfatal ischemic stroke and all-cause mortality when compared to clopidogrel, however, significantly increased bleeding events in Chinese patients with ACS and severe renal insufficiency undergoing PCI. Large-scale, long-term, randomized trials should be required to find the efficacy and safety dose of ticagrelor in East Asian patients with ACS and severe renal insufficiency.

## Figures and Tables

**Table 1:** Baseline characteristics of the study participants.

Variables	Total (*n* = 276)	Ticagrelor (*n* = 108)	Clopidogrel (n = 168)	*P* value
Male, no.(%)	192 (69.6)	79 (73.1)	113 (67.4)	0.473

Age(years),mean ± SD	67.5 ± 11.6	66.3 ± 10.8	68.2 ± 12.1	0.24

Diagnosis at discharge, *n* (%)				0.085
STEMI	117 (42.4)	48 (44.4)	69 (41.1)	
NSTEMI	86 (31.2)	40 (37.0)	46 (27.4)	
Unstable angina	73 (26.4)	20(18.5)	53 (31.5)	

Risk factors, *n* (%)				
Hypertension	210 (76.1)	78 (72.2)	132 (78.6)	0.367
Diabetes	109 (39.5)	36 (33.3)	73 (43.5)	0.187
Current smoker	70 (25.4)	32 (29.6)	38 (22.6)	0.326

Previous medical history, *n* (%)				
Myocardial infarction	28 (10.1)	12(11.1)	16 (9.5)	0.798
Congestive heart failure	63 (22.8)	20 (18.5)	43 (25.6)	0.289
COPD	20 (6.9)	5 (4.6)	15 (8.9)	0.204
Nonhemorrhagic stroke	22 (10.2)	14 (12.9)	16 (9.5)	0.466

Dialysis, *n* (%)	50 (18.1)	18 (16.7)	32 (19.0)	0.83

SBP (mmHg), mean ± SD	134.8 ± 31.5	132.9 ± 30.9	135.9 ± 31.9	0.494

DBP (mmHg), mean ± SD	79.5 ± 16.4	78.6 ± 16.1	80.0 ± 16.5	0.57

HR (bpm), mean ± SD	86.3 ± 14.6	87.1 ± 13.4	85.9 ± 15.3	0.566

Hemoglobin (g/L), mean ± SD	106.2 ± 14.4	108.3 ± 10.4	105.0 ± 16.1	0.104

eGFR (ml/min·1.73 m^2^), median (IQR)	17.3 (10.6, 24.3)	18.0 (12.3, 24.4)	17.0 (9.1, 24.2)	0.281

Creatinine (*μ*mol/L), mean ± SD	404.2 ± 267.4	369.0 ± 227.9	424.1 ± 286.3	0.147

Hs-cTn T (pg/mL), Mean ± SD	1544.5 ± 2497.3	1263.4 ± 2095.7	1703.3 ± 2692.3	0.214

CK-MB (U/L), Mean ± SD	137.5 ± 192.2	130.4 ± 180.0	141.4 ± 199.4	0.688

NT-proBNP(pg/mL), mean ± SD	16372.1 ± 11914.7	14931.3 ± 11720.8	17186.5 ± 11988.4	0.182

EF (%), mean ± SD	50.4 ± 6.8	50.5 ± 7.0	50.4 ± 6.8	0.936

BMI (kg/m^2^), mean ± SD	23.8 ± 2.2	24.1 ± 2.2	23.7 ± 2.1	0.155

Cholesterol (mmol/L),mean ± SD	4.3 ± 1.1	4.4 ± 1.3	4.2 ± 1.0	0.217

LDL-C (mmol/L), mean ± SD	2.4 ± 1.0	2.5 ± 1.1	2.3 ± 0.9	0.248

Glucose (mmol/L), mean ± SD	8.2 ± 3.3	8.3 ± 3.2	8.1 ± 3.3	0.808

Radial access, *n* (%)	265 (96.0)	104 (96.3)	161 (95.8)	0.714

Number of diseased vessels, *n* (%)				0.144
One	43 (15.6)	19 (17.6)	24 (14.3)	
Two	115 (41.6)	36 (33.3)	79 (47.0)	
Three	118 (42.8)	53 (49.1)	65 (38.7)	

Complete revascularization, *n* (%)	182 (66.0)	78 (72.2)	102 (62.0)	0.387

Medication on arrival, *n* (%)				
Aspirin	276 (100.0)	108 (100)	168 (100)	1
GP IIb/IIIa inhibitors	55 (19.9)	28 (25.9)	27 (16.1)	0.121
LMWH	131 (47.5)	47 (43.5)	84 (50.0)	0.445
PPI	215 (77.9)	87 (80.6)	128 (76.1)	0.532
*β*-blocker	232 (84.1)	95 (88.0)	137 (81.5)	0.28
ACE inhibitor and/or ARB	142 (51.4)	56 (51.9)	86 (51.2)	0.987
Stain, *n* (%)	276 (100.0)	108 (100)	168 (100)	1
CCB, *n* (%)	152 (55.1)	58 (53.7)	94 (56.0)	0.752

STEMI, ST-elevation myocardial infarction; NSTEMI, non-ST-elevation myocardial infarction; COPD, chronic obstructive pulmonary disease; SBP, systolic blood pressure; DBP, diastolic blood pressure; HR, heart rate; eGFR, estimated glomerular filtration rate calculated by the chronic kidney disease epidemiology equation; hs-cTn T, high sensitivity cardiac troponin T; CK-MB, heart-type isoenzyme of creatine kinase; NT-proBNP, N-terminal pro-brain natriuretic peptide; EF, ejection fraction; LDL-C, low-density lipoprotein cholesterol; LMWH, low-molecular-weight heparin; PPI, proton pump inhibitors; CCB, calcium channel blockers.

**Table 2:** The association between use of different antiplatelet agents and cardiovascular outcomes using an extended cox model.

Outcome	Events (*n*)		Nonadjusted model		Model I		Model 2	
	Ticagrelor (*n* = 108)	Clopidogrel (*n* = 168)	HR (95% CI)	*P* value	HR (95% CI)	*P* value	HR (95% CI)	*P* value
Primary end point (composite of CV death, MI and stroke)	39 (36.1)	69 (41.1)	0.83 (0.56∼1.23)	0.353	0.81 (0.50∼1.28)	0.378	0.78 (0.46∼1.33)	0.367
Death from any cause	21 (19.4)	38 (22.6)	0.83 (0.45∼1.55)	0.563	0.86 (0.47∼1.61)	0.645	0.86 (0.38∼1.89)	0.708
Cardiovascular death	15 (13.9)	30 (17.9)	0.72 (0.35∼1.46)	0.356	0.73 (0.36∼1.49)	0.385	0.80 (0.35∼2.12)	0.521
Myocardial infarction	10 (9.3)	23 (13.7)	0.59 (0.25∼1.4)	0.231	0.59 (0.25∼1.4)	0.233	0.72 (0.31∼2.36)	0.485
Ischemic stroke	8 (7.4)	13 (7.7)	0.91 (0.34∼2.46)	0.852	0.94 (0.35∼2.57)	0.909	0.46 (0.24∼3.07)	0.172

**Table 3:** The association between use of different antiplatelet agents and bleeding outcomes using an extended cox model.

Outcome	Events (*n*)		Nonadjusted model		Model I		Model 2	
	Ticagrelor (*n* = 108)	Clopidogrel (*n* = 168)	HR (95% CI)	*P* value	HR (95% CI)	*P* value	HR (95% CI)	*P* value
Total BARC bleedings	39 (36.1)	37 (22.0)	1.88 (1.12∼3.15)	0.026	1.96 (1.17∼3.28)	0.021	3.01 (1.81∼5.62)	0.010
BARC 1 or 2 bleedings	25 (23.1)	24 (14.3)	1.83 (0.97∼3.46)	0.064	2.06 (1.08∼3.92)	0.038	3.14 (1.52∼5.76)	0.018
BARC 3 or 5 bleedings	14 (13.0)	13 (7.7)	1.98 (0.83∼4.77)	0.116	1.9 (0.79∼4.56)	0.101	2.87 (1.12∼7.03)	0.045

*Notes.* data presented are HRs and 95% CIs. Adjust I model adjusts for age and gender; adjust II model adjust for variables that, when added to this model, changed the matched odds ratio by at least 10 percent, including adjusting for adjust I + smoking status, diabetes, diagnosis, congestive heart failure, COPD, stroke history, heart rate, hemoglobin, ejection fraction, hs-cTn T, Nt-proBNP, eGFR, dialysis, GP IIb/IIIa inhibitors, 3-vessel disease.

## Data Availability

The datasets generated and analyzed during the current study are available from the corresponding author on reasonable request.
